# Comparison of Cytokines Profiles and Monocyte Response Among Tuberculosis Patients Versus Patients Coinfected With Intestinal Helminth and Tuberculosis

**DOI:** 10.7759/cureus.51726

**Published:** 2024-01-05

**Authors:** Ritu Kumari, Sweta Muni, Randhir Kumar, Rakesh Kumar, Abhay Kumar, Shailesh Kumar, Namrata Kumari

**Affiliations:** 1 Microbiology, Indira Gandhi Institute of Medical Sciences, Patna, IND

**Keywords:** cytokines, helminths, interferon-gamma, interleukins, monocytes, tuberculosis

## Abstract

Background

Tuberculosis (TB) and intestinal helminth infections often coexist, posing a significant health challenge. TB, caused by *Mycobacterium tuberculosis*, and helminths elicit distinct immune responses - Th1 for TB and Th2 for helminths. Co-infection introduces a complex immunological challenge, potentially compromising TB control. This study addresses the research gap by comparing cytokine profiles and monocyte responses in TB patients, helminth-infected individuals, and those with both. Insights gained may enhance diagnosis, treatment, and disease control strategies where TB and helminths prevail.

Methods

A cross-sectional observational study conducted at Indira Gandhi Institute of Medical Sciences, Patna, Bihar, aimed to compare cytokine profiles and monocyte responses in TB patients and those coinfected with TB and helminths. The study included 150 newly diagnosed active TB individuals aged 18 to 65 years. TB diagnosis was confirmed through clinical assessment, sputum microscopy, and GeneXpert (Cepheid, Sunnyvale, CA, USA) testing. Stool examination employed various methods, including the Kato-Katz technique and formalin-ether concentration. Blood samples were collected for hematological analysis, cytokine profiling, and monocyte isolation. Statistical analysis, using SPSS version 20.0 (IBM Corp., Armonk, NY, USA), included descriptive statistics, and t-test analyses.

Results

In our study of 150 participants, half (50.0%) showed positive helminth status. The sociodemographic analysis revealed no significant differences in age, gender, education, occupation, marital status, smoking, alcohol, BMI, diabetes, and hypertension between TB patients (n=75) and TB+Helminth patients (n=75), ensuring baseline matching. The prevalence of specific helminth infections in TB+Helminth patients included *Ascaris lumbricoides* (24.0%),* Trichuris trichiura* (18.7%), and others. Hematological parameters showed significant differences, with TB+Helminth patients exhibiting higher RBC count, hemoglobin, hematocrit, neutrophil count, and monocyte count; also eosinophil count was more raised in TB+Helminth patients (0.36 x 10^3^/μL) when compared to TB patients (0.25 x 10^3^/μL). Cytokine profiles and monocyte responses varied significantly between the groups, with TB patients having higher IL-4, IL-6, IFN-γ, TNF-α, and IL-1β levels, while TB+Helminth patients had elevated IL-10. Monocyte response time did not differ significantly.

Conclusion

The observed differences in hematological parameters and cytokine profiles emphasize the need for tailored approaches to diagnosis and treatment in co-infected individuals. These findings suggest that the management of TB patients should consider the potential influence of helminth co-infections.

## Introduction

Tuberculosis (TB) and intestinal helminth infections are two widespread global health challenges that have historically been studied in isolation [1]. In 2020, approximately 10 million new cases of tuberculosis (TB) were reported and in India, the cases increased by 19% as compared to the previous year. It is believed that around 2 billion people, constituting about one-quarter of the global population, are harboring latent infections by Mycobacterium tuberculosis (Mtb) and in India, it is estimated that 40% of the population is infected with Tb bacteria. Among these individuals, 5-10% are anticipated to progress to active TB (ATB) [2]. The infections attributable to soil-transmitted helminths (STH) and schistosomes are prevalent, affecting approximately 1.5 billion and 250 million people globally, respectively [3]. However, their co-occurrence in many regions around the world has raised important questions about the impact of co-infection on the host's immune response [4]. While TB remains a leading cause of morbidity and mortality globally, intestinal helminths, including various species of nematodes, trematodes, and cestodes, collectively infect billions of individuals, particularly in resource-limited areas (including poor sanitation, overcrowded living conditions, lack of access to clean water, lack of proper hygiene infrastructure, and socioeconomic disparities) [4].

Understanding the interactions between these two distinct infectious agents is essential for both public health and the advancement of immunological knowledge. The immune response to TB is a finely orchestrated interplay of pro-inflammatory and anti-inflammatory cytokines, orchestrated by immune cells such as monocytes and macrophages [5,6]. Effective containment of M. tuberculosis depends on the activation of Th1-type immune responses, characterized by the production of cytokines like interferon-gamma (IFN-γ) and tumor necrosis factor-alpha (TNF-α). In contrast, intestinal helminths typically induce a Th2-type immune response, characterized by the release of interleukin-4 (IL-4), interleukin-5 (IL-5), and interleukin-13 (IL-13), which promote an anti-inflammatory environment [7,8].

The co-infection scenario, where intestinal helminths also afflict TB patients, introduces a complex immunological conundrum [9]. The immunomodulatory effects of helminths are well-documented, and their capacity to skew the immune response towards a Th2 phenotype may potentially undermine the host's ability to mount an effective Th1 response against TB [10]. This interaction has significant implications for the clinical management of both diseases and the design of public health interventions, as co-infection can potentially exacerbate disease severity, hinder diagnosis, and impact treatment outcomes [11].

However, there is a paucity of research that directly compares the immune responses of individuals with TB to those co-infected with both TB and intestinal helminths, as well as those solely afflicted by helminths [11]. To address this gap in knowledge, this study aimed to compare cytokine profiles and monocyte responses among these distinct patient groups. This study is not only pivotal for understanding the complex immunological dynamics within co-infected individuals but also holds the potential to inform strategies for improved diagnosis, treatment, and disease control in regions where TB and intestinal helminths commonly coexist.

## Materials and methods

Study design

This cross-sectional observational study was conducted at Indira Gandhi Institute of Medical Sciences, Patna, Bihar for a period of six months between January to June 2023 in the Department of Microbiology among patients suffering from pulmonary tuberculosis. Ethical approval was obtained from the Institutional Ethics Committee [Letter number: 830/IEC/IGIMS/2022], and written informed consent was obtained from all participants.

Study participants

The study included 150 individuals aged 18 to 65 years, newly diagnosed with active TB, during a defined study period. TB diagnosis was confirmed by clinical assessment, sputum microscopy, and GeneXpert (Cepheid, Sunnyvale, CA, USA) testing. Exclusion criteria were defined to exclude patients with known immunodeficiency, including those with HIV-positive status. Blood sugar levels were assessed, and individuals with uncontrolled diabetes were excluded. Detailed drug history, particularly immunosuppressive medications, was considered, and participants on such drugs were excluded. The use of alternative medicine, especially those containing steroids, was also grounds for exclusion to ensure a more homogeneous study population with respect to immunologic responses. Also, individuals on antitubercular drugs and anti-parasitic drugs, and pregnant patients were excluded from the study.

Stool examination

Participants with TB underwent a comprehensive stool examination for the presence of intestinal helminths. The examination included both macroscopic and microscopic assessments. Macroscopic examination of the fecal sample was conducted to assess color, consistency, and the presence of blood. For the microscopic evaluation, a combination of methods was employed to ensure the thorough detection of helminths and protozoa.

Kato-Katz Technique

The Kato-Katz technique was used, involving the preparation of a smear on a microscope slide. This smear was examined for the presence of helminth eggs or larvae.

Direct Wet Mount Method

Additionally, a saline-wet mount and iodine mount preparations were prepared from the fecal samples. These preparations were observed under a light microscope at 10X magnification.

Formalin-Ether Concentration Method

The formalin-ether concentration method was utilized to further enhance the detection of helminth ova and larvae. After concentration, the samples were examined under a light microscope at 10X and further confirmed by observation at 40X magnification. This method aimed to improve the sensitivity of helminth detection.

The stool examination was performed by experienced laboratory technicians who followed established protocols and quality control measures to ensure the accurate and thorough detection of intestinal helminths, as well as the presence of any protozoa in the samples.

Sample collection

Blood samples (10 mL) were collected from all participants in sterile ethylenediamine tetraacetic acid (EDTA) vacutainers for the analysis of hematological parameters, cytokines and monocyte responses. The hematology analyzer (Sysmex XP-100, Sysmex, Singapore), facilitated the simultaneous examination of various blood components, including red blood cells (RBCs), white blood cells (WBCs), platelets, hemoglobin levels, and hematocrit.

Cytokine profiling

The processing of serum samples was expedited, with a strict time limit of within 2 hours from collection to ensure sample integrity. The study focused on measuring the concentrations of key cytokines, namely interleukin-4 (IL-4), interleukin-6 (IL-6), interleukin-10 (IL-10), interferon-gamma (IFN-γ), and tumor necrosis factor-alpha (TNF-α). These measurements were achieved through the utilization of flow cytometer (BD Accuri™ C6 Plus Flow Cytometer, Becton Dickinson, Franklin Lakes, NJ, USA), in strict accordance with the manufacturer's instructions. Flow cytometer, a highly precise and reliable method, allowed us to quantify the concentrations of these cytokines in the collected serum samples.

Monocyte isolation and activation

Initially, peripheral blood mononuclear cells (PBMCs) were isolated from the collected blood samples. This isolation was achieved through a highly controlled and precise method known as density gradient centrifugation. It allowed for the efficient separation of PBMCs, critical components of the immune system, from the whole blood. Monocytes were subsequently isolated from the isolated PBMCs. This was achieved using CD14+ magnetic bead separation, a method known for its accuracy and reliability. CD14+ is a specific surface marker found on monocytes, enabling their targeted isolation. Isolated monocytes were then cultured carefully in a selected medium, RPMI-1640, supplemented with 10% fetal bovine serum (FBS). To assess their pro-inflammatory capabilities, these monocytes were stimulated with lipopolysaccharide (LPS). LPS is a potent activator of the immune response and was used in this study to trigger the release of pro-inflammatory cytokines. The pro-inflammatory response was assessed by quantifying key cytokines, namely interleukin-6 (IL-6) and interleukin-12 (IL-12), recognized for their central roles in driving inflammatory immune reactions.

Data collection

A detailed pre-validated questionnaire was meticulously designed, incorporating questions that covered a wide range of sociodemographic variables (age, gender, educational background, occupation, and marital status) and other relevant details (dietary habits, lifestyle factors, any known exposure to risk factors relevant to TB and helminth infections, clinical and medical history, any existing medical conditions).

Data analysis

The study participants were divided into two distinct groups: group A, comprising exclusively TB patients, and group B, consisting of patients who were coinfected with both TB and Helminths. The statistical analysis was performed using SPSS version 20.0 (IBM Corp., Armonk, NY, USA). Descriptive statistics were employed to summarize the demographic characteristics of the study participants. The data type for this analysis was categorical (frequency and percentage) and continuous (mean and SD). Hematological parameters, cytokine profiles and monocyte responses were compared between the two distinct groups: TB patients and TB+Helminth coinfected individuals. These parameters were continuous data, and the comparison was conducted using Student’s t-tests. Throughout the analysis, statistical significance was considered at a predefined alpha (0.05) level, with p-values less than 0.05 considered statistically significant.

Ethical considerations

The study followed the principles of the Declaration of Helsinki. Informed consent was obtained from all participants, and data were anonymized to ensure confidentiality. The research adhered to the ethical guidelines outlined in the approved protocol.

## Results

Our study included 150 participants, with 50.0% (n=75) testing positive for helminth infections on stool examination. The cohort comprised 75 patients with TB alone and 75 with both TB and helminth infections. Sociodemographic analysis revealed no significant age differences between TB patients (45.6 ± 6.3 years) and those with TB+Helminth infections (46.2 ± 6.1 years) (p = 0.554). Gender distribution was comparable, with 60.0% males (45/75) in the TB group and 56.0% (42/75) in the TB+Helminth group (p = 0.619). Education levels, predominantly up to secondary and higher secondary schooling, showed no significant intergroup differences (p = 0.920). Occupation, marital status, smoking history, alcohol consumption, body mass index (BMI), and comorbidities (diabetes and hypertension) also exhibited no significant discrepancies, ensuring baseline comparability (Table 1).

**Table 1:** Sociodemographic Characteristics of Study Participants.

Characteristic	TB Patients (N=75) Number (%)/ Mean ± SD	TB+Helminth Patients (N=75) Number (%)/ Mean ± SD	p-value
Age (years)	45.6 ± 6.3	46.2 ± 6.1	0.554
Gender			
Male	45 (60.0%)	42 (56.0%)	0.619
Female	30 (40.0%)	33 (44.0%)	
Education Level			
Below Primary	10 (13.3%)	12 (16.0%)	0.920
Primary and middle school	25 (33.4%)	22 (29.3%)	
Secondary and higher secondary school	30 (40.0%)	32 (42.7%)	
Graduate and above	10 (13.3%)	9 (12.0%)	
Occupation			
Unemployed	12 (16.0%)	11 (14.7%)	0.979
Unskilled and semiskilled	30 (40.0%)	29 (38.7%)	
Skilled	15 (20.0%)	17 (22.6%)	
Professional or semi-professional	18 (24.0%)	18 (24.0%)	
Marital Status			
Unmarried	25 (33.3%)	27 (36.0%)	0.937
Married	40 (53.4%)	38 (50.7%)	
Divorced and widowed	10 (13.3%)	10 (13.3%)	
Tobacco/Smoking History			
No	55 (73.4%)	58 (77.3%)	0.837
Former	10 (13.3%)	9 (12.0%)	
Current	10 (13.3%)	8 (10.7%)	
Alcohol Consumption			
Non-drinkers	60 (80.0%)	57 (76.0%)	0.839
Moderate Drinkers	10 (13.3%)	12 (16.0%)	
Heavy Drinkers	5 (6.7%)	6 (8.0%)	
Body Mass Index (BMI) (kg/m^2^)	21.8 ± 3.4	21.5 ± 3.2	0.578
Comorbidities			
Diabetes	12 (16.0%)	11 (14.7%)	0.820
Hypertension	18 (24.0%)	17 (22.7%)	0.846

TB+Helminth patients had diverse infections: Ascaris lumbricoides (24.0%), Trichuris trichiura (18.7%), hookworms (12.0%), Strongyloides stercoralis (9.3%), Enterobius vermicularis (5.3%), Taenia spp. (8.0%), and Schistosoma spp. (6.7%), each with distinct symptoms. Various other helminths affected 16.0% of patients, emphasizing the need to consider their diverse clinical manifestations (Table 2).

**Table 2:** Prevalence of Specific Helminth Infections in TB+Helminth Patients.

Helminth Species	Frequency	%	Clinical Symptoms
Ascaris lumbricoides	18	24.0%	Abdominal pain, diarrhea (loose or watery stools)
Trichuris trichiura	14	18.7%	Abdominal discomfort, anemia (microcytic and hypochromic)
Hookworm (Ancylostoma duodenale or Necator americanus)	9	12.0%	Iron-deficiency anemia, gastrointestinal bleeding
Strongyloides stercoralis	7	9.3%	Abdominal pain, skin rash
Enterobius vermicularis	4	5.3%	Perianal itching, restless sleep
Taenia spp. (tapeworm)	6	8.0%	Nausea, weight loss
Schistosoma spp.	5	6.7%	Abdominal pain, blood in urine
Other Helminths	12	16.0%	-

In the evaluation of hematological parameters between TB patients (n=75) and TB+Helminth patients (n=75), significant differences emerged. TB+Helminth patients demonstrated higher Red Blood Cell Count, Hemoglobin levels, and Hematocrit percentage compared to TB patients (p < 0.0001, p = 0.0001, p = 0.048, respectively). Additionally, TB+Helminth patients displayed elevated Neutrophil Count and Monocyte Count (p = 0.023, p < 0.0001, respectively). However, Mean Corpuscular Volume, White Blood Cell Count, Lymphocyte Count, and Platelet Count showed no significant differences between the two groups (Table 3).

**Table 3:** Comparison of Hematological Parameters in TB and TB+Helminth Patients.

Hematological Parameter	TB Patients (n=75) Mean ± SD	TB+Helminth Patients (n=75) Mean ± SD	p-value
Red Blood Cell Count (x10^6^/μL)	4.5 ± 0.3	4.8 ± 0.9	< 0.0001
Hemoglobin (g/dL)	11.3 ± 1.2	12.2 ± 1.6	0.0001
Hematocrit (%)	39.1 ± 2.0	39.8 ± 2.3	0.048
Mean Corpuscular Volume (fL)	88.2 ± 4.5	88.5 ± 5.2	0.706
White Blood Cell Count (x10^3^/μL)	7.5 ± 2.0	7.8 ± 2.1	0.371
Neutrophil Count (x10^3^/μL)	4.3 ± 1.0	4.8 ± 1.6	0.023
Lymphocyte Count (x10^3^/μL)	2.8 ± 0.9	2.7 ± 0.8	0.473
Monocyte Count (x10^3^/μL)	0.5 ± 0.2	0.7 ± 0.2	<0.0001
Platelet Count (x10^3^/μL)	248.1 ± 41.3	258.6 ± 43.2	0.130
Eosinophil count (x10^3^/μL)	0.25 ± 0.13	0.36 ± 0.14	< 0.0001

In analyzing cytokine profiles and monocyte responses, distinct differences emerged between TB patients (n=75) and TB+Helminth patients (n=75). TB patients displayed higher levels of Interleukin-4 (IL-4), Interleukin-6 (IL-6), Interferon-gamma (IFN-γ), and Tumor Necrosis Factor-alpha (TNF-α) compared to TB+Helminth patients. Monocyte pro-inflammatory cytokines (IL-1β) were significantly elevated in TB patients, while Monocyte Anti-Inflammatory Cytokines (IL-10) were higher in TB+Helminth patients. Monocyte response time did not differ significantly between the groups. Other cytokines, including IL-5, IL-10, IL-12, IL-17, IL-18, and Monocyte Pro-Inflammatory Cytokines (IL-8, IL-13), showed no significant differences (Table 4).

**Table 4:** Comparison of Cytokine Profiles and Monocyte Responses in TB and TB+Helminth Patients. *Measuring unit as pg/mL

Cytokine/Monocyte Response*	TB Patients (n=75) Mean ± SD	TB+Helminth Patients (n=75) Mean ± SD	p-value
Interleukin-4 (IL-4)	13.7 ± 2.5	12.5 ± 2.0	0.001
Interleukin-5 (IL-5)	9.1 ± 1.7	8.8 ± 1.3	0.226
Interleukin-6 (IL-6)	27.3 ± 4.9	25.6 ± 4.2	0.024
Interleukin-10 (IL-10)	15.3 ± 3.1	14.7 ± 2.9	0.222
Interferon-gamma (IFN-γ)	30.8 ± 3.8	28.9 ± 3.5	0.002
Tumor Necrosis Factor-alpha (TNF-α)	20.9 ± 3.8	18.3 ± 2.7	<0.0001
Interleukin-12 (IL-12)	11.4 ± 1.9	11.2 ± 1.8	0.509
Interleukin-17 (IL-17)	9.6 ± 1.6	9.4 ± 1.4	0.416
Interleukin-18 (IL-18)	13.5 ± 2.3	13.1 ± 2.1	0.267
Monocyte Pro-Inflammatory Cytokines (IL-1β)	6.3 ± 1.1	5.2 ± 1.0	<0.0001
Monocyte Pro-Inflammatory Cytokines (IL-8)	7.3 ± 1.4	7.1 ± 1.3	0.366
Monocyte Anti-Inflammatory Cytokines (IL-13)	3.6 ± 0.9	3.8 ± 1.1	0.224
Monocyte Anti-Inflammatory Cytokines (IL-10)	4.9 ± 1.2	5.5 ± 1.9	0.022
Monocyte Response Time (hours)	24.0 ± 2.5	23.8 ± 2.6	0.631

## Discussion

The interplay between TB and helminth infections has raised questions about the potential impact of co-infections on various clinical, hematological, and immunological aspects. In our study, we sought to shed light on these interactions and their implications for patient outcomes. In our study, a total of 150 participants were enrolled and half of them (50.0%) had positive helminth status on stool examination (n=75). A similar prevalence of 50.0% or more was observed in the studies by Brown et al., Elias et al., and Tristão-Sá et al. [12-14]. In contrast, studies by Abbas et al. and Resende et al. showed a lower prevalence of helminth infection among TB patients [15,16].

Our investigation began by comparing the sociodemographic and clinical characteristics of TB patients with and without helminth co-infections. Importantly, the study groups were found to be well-matched at baseline, as we observed no significant differences in age, gender distribution, education levels, occupation, marital status, smoking history, alcohol consumption, or body mass index (BMI) between the two groups. Furthermore, comorbidities, such as diabetes and hypertension, were similarly prevalent in both cohorts. In our study, in terms of education, the majority of participants in both groups had received education up to the secondary and higher secondary school level. The distribution of education levels did not differ significantly between the two groups (p = 0.920). But studies by Moraes Neto et al. and Belkaid et al. have shown a higher prevalence of helminth among lower education levels [17,18].

This baseline comparability is crucial for ensuring that any observed differences in clinical, hematological, and immunological parameters can be attributed to the presence or absence of helminth infections, rather than pre-existing disparities in the study populations. The absence of significant differences in these sociodemographic and clinical factors reassures the validity of our subsequent findings.

A critical aspect of our study involved the examination of cytokine profiles and monocyte responses in TB and TB+Helminth patients. Notably, we observed differences in several cytokines between the two groups, with higher levels of Interleukin-4 (IL-4) in TB patients. IL-4 is associated with Th2-type immune responses and plays a role in mediating allergic and anti-helminth immune responses. This finding suggests that TB patients may exhibit a more pronounced Th2-type response, possibly as a result of concurrent helminth infections [8,9].

Conversely, the levels of several cytokines, including Interleukin-6 (IL-6), Interferon-gamma (IFN-γ), and Tumor Necrosis Factor-alpha (TNF-α), were elevated in TB patients, indicating a potentially heightened pro-inflammatory response. This may be related to the immune activation associated with TB. In our study, Interferon-gamma (IFN-γ) was notably elevated in TB patients, with a mean of 30.8 ± 3.8 pg/mL, in contrast to TB+Helminth patients with a mean of 28.9 ± 3.5 pg/mL (p = 0.002). A similar pattern was observed in the studies by Elias et al., Abbas et al., Resende et al., Babu et al., Du Plessis et al., Cadmus et al., and Anuradha et al. [13,15,16,19-22].

In our study, Interleukin-4 (IL-4) was significantly higher in TB patients, with a mean of 13.7 ± 2.5 pg/mL, as compared to TB+Helminth patients, who exhibited a lower mean of 12.5 ± 2.0 pg/mL (p = 0.001). Interleukin-6 (IL-6) levels were significantly higher in TB patients, with a mean of 27.3 ± 4.9 pg/mL, compared to TB+Helminth patients, who had a mean of 25.6 ± 4.2 pg/mL (p = 0.024). Similar increased IL-4 and IL-6 were raised in the TB group in studies by Elias et al., Adams et al., Wang et al., Monin et al., and Potian et al. [13,23-26].

In our study, other cytokines, including Interleukin-10 (IL-10), Interleukin-12 (IL-12), Interleukin-17 (IL-17), and Interleukin-18 (IL-18), showed no significant differences between the two groups, whereas studies by Rook; Boneberg and Hartung; Santiago et al.; Lang and Schick; and Rajamanickam et al. have shown higher levels in Interleukin-10 (IL-10), Interleukin-12 (IL-12), in helminths+TB groups and the difference was statistically significant [27-31].

An intriguing finding was the significantly higher levels of Monocyte Pro-Inflammatory Cytokines (IL-1β) in TB patients. This observation underscores the complexity of monocyte responses in the context of TB and helminth co-infections, as it suggests a more pro-inflammatory environment in TB patients. In contrast, studies by Bazzone et al., Waitt et al., Feske et al., and Waitt et al. have also shown lower levels of Monocyte Pro-Inflammatory Cytokines (IL-1β) in TB patients [32-35].

Additionally, Monocyte Anti-Inflammatory Cytokines (IL-10) were significantly higher in TB+Helminth patients. IL-10 is known for its immunosuppressive properties and can modulate both Th1 and Th2 responses. The elevated IL-10 levels in TB+Helminth patients may reflect an attempt to regulate the immune response in the face of co-infections.

Collectively, these findings indicate that helminth co-infections may modulate the immune response and contribute to complex interactions in co-infected individuals. These interactions may involve a delicate balance between pro-inflammatory and anti-inflammatory pathways, with potential implications for disease progression and treatment outcomes.

Limitations

The limitations of our study should be acknowledged. It was conducted in a specific geographic region, and the prevalence of helminth species may vary in other areas. Additionally, the study's cross-sectional design limits our ability to establish causality. Longitudinal studies and in-depth mechanistic investigations are needed to elucidate the complex interactions between TB and helminths.

## Conclusions

In conclusion, our study highlights the intricate relationships between helminth infections and TB, affecting various aspects of clinical, hematological, and immunological parameters. The observed differences in hematological parameters and cytokine profiles emphasize the need for tailored approaches to diagnosis and treatment in co-infected individuals. These findings suggest that the management of TB patients should consider the potential influence of helminth co-infections. Further research is needed to unravel these co-infections' underlying mechanisms and clinical outcomes. By better understanding the interplay between TB and helminths, we can develop more effective strategies for diagnosing, treating, and preventing these co-occurring diseases. As we move forward, we must recognize that infectious diseases rarely occur in isolation. They are often intertwined in complex ways that demand a holistic approach to patient care and research. Our study contributes to this broader understanding and underscores the importance of addressing co-infections as a critical aspect of global health research and practice.
